# Conjoined twins in dichorionic diamniotic triplet pregnancy: a report of three cases and literature review

**DOI:** 10.1186/s12884-021-04165-x

**Published:** 2021-10-08

**Authors:** Hongyan Liu, Chunyan Deng, Qing Hu, Hua Liao, Xiaodong Wang, Haiyan Yu

**Affiliations:** 1 Department of Obstetrics and Gynecology, West China Second University Hospital, Sichuan University, Chengdu, China; 2 Key Laboratory of Birth Defects and Related Diseases of Women and Children (Sichuan University), Ministry of Education, No. 20, 3rd section, South Renmin Road, Chengdu, 610041 Sichuan China

**Keywords:** Conjoined twins, Dichorionic diamniotic triplet pregnancy, Selective termination, Literature review

## Abstract

**Background:**

Conjoined twins are a rare and serious complication of monochorionic twins. The total incidence is 1.5 per 100,000 births, and about 50% are liveborn. Prenatal screening and diagnosis of conjoined twins is usually performed by ultrasonography. Magnetic resonance imaging can be used to assist in the diagnosis if necessary. Conjoined twins in dichorionic diamniotic triplet pregnancy are extremely rare.

**Case presentation:**

We reported three cases of dichorionic diamniotic triplet pregnancy with conjoined twins. Due to the poor prognosis of conjoined twins evaluated by multidisciplinary teams, selective termination of conjoined twins was performed in three cases. In case 1, selective reduction of the conjoined twins was performed at 16 gestational weeks, and a healthy female baby weighing 3270 g was delivered at 37 weeks. In case 2, the conjoined twins were selectively terminated at 17 weeks of gestation, and a healthy female baby weighing 2760 g was delivered at 37 weeks and 4 days. In case 3, the conjoined twins were selectively terminated at 15 weeks and 2 days, and a healthy female baby weighing 2450 g was delivered at 33 weeks and 6 days. The babies of all three cases were followed up and are in good health.

**Conclusion(s):**

Surgical separation is the only treatment for conjoined twins after birth. Early determination of chorionicity and antenatal diagnosis of conjoined twins in triplet gestations are critical for individualized management options and the prognosis of normal triplets. Expecting parents should be extensively counseled by multidisciplinary teams. If there are limitations in successful separation after birth, early selective termination of the conjoined twins by intrathoracic injection of potassium chloride may be a procedure in dichorionic diamniotic triplet pregnancy to improve perinatal outcomes of the normal triplet.

## Background

Conjoined twins are a rare and serious complication of monochorionic twins. The total incidence is 1.5 per 100,000 births, and about 50% are liveborn [1]. The survival rate of conjoined twins is low, and the prognosis is generally poor. Common triplet pregnancies are monochorionic triamniotic, trichorionic triamniotic, and dichorionic triamniotic, and only 2% are dichorionic diamniotic triplet pregnancies [2]. Conjoined twins in a triplet pregnancy are rare, and the incidence is less than one in a million deliveries [3]. Conjoined twins in a dichorionic diamniotic (DCDA) triplet pregnancy are extremely rare. So far, there are only a few published articles relevant to conjoined twins in triplet pregnancies, and the majority of them are case reports. We have only found 10 cases of conjoined twins reported in DCDA triplet pregnancies.

Here, we reported three cases of conjoined twins in dichorionic diamniotic triplet pregnancies, in which selective fetal reduction by intracardiac injection of potassium chloride was performed. As a result, another fetus continued to grow and develop in the uterus and was delivered at full term, which was a good pregnancy outcome. Additionally, we used a list of keywords including “conjoined twins”, “triplets,” “triplet pregnancy”, and “multiple pregnancy” to perform an extensive Medline search and conducted a literature review in English and Chinese about conjoined twins in triplet pregnancies. Written informed consent was obtained from the couples before the procedure and manuscript publication. The treatment procedure followed ethical principles, and all data was collected from chart reviews. This study was approved by the ethical committees at the West China Second University Hospital of Sichuan University.

## Case presentation

### Case 1

**Fig. 1:**
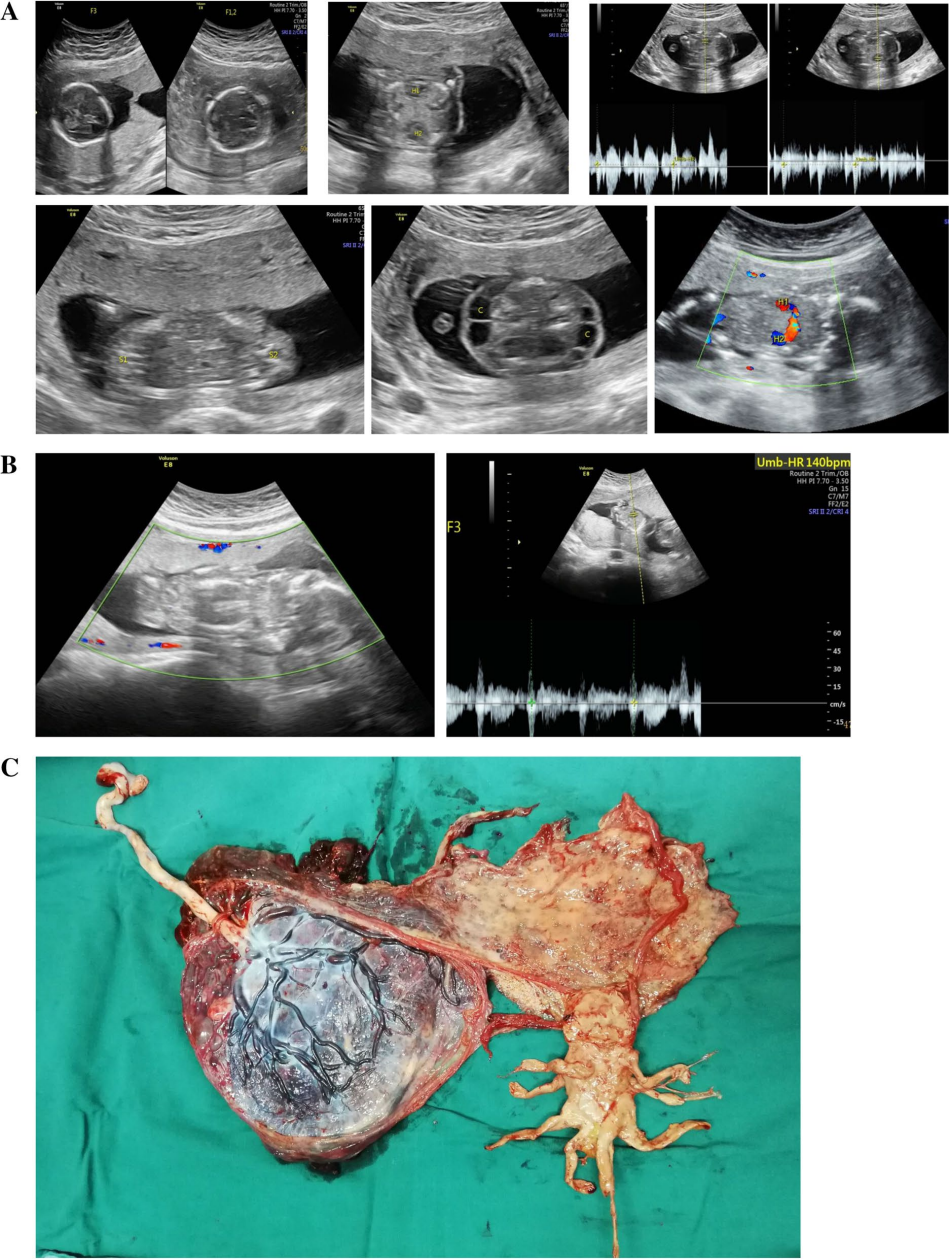
**A.** Images of conjoined twins (F1 and F2) of case 1. **B.** Images of live fetus (F3) and conjoined twins after selective termination of case 1. **C.** Images of the dichorionic-diamniotic placenta and the papyraceous conjoined twins of case 1

A 44-year-old woman, gravida 4, para 1, spontaneously conceived. Ultrasound examination at 12 weeks of gestation showed cranio-thoraco-omphalopagus conjoined twins in a DCDA triplet pregnancy. In the conjoined twins, there was only one skull halo, two sets of thalamus and cerebellum, and partial fusion of frontal brain tissue of two fetuses. Partial fusion of the neck, which segregated cystic space, was seen in both fetal necks, as well as a cystic mass measuring 2.9 × 1.9 cm and 2.8 × 1.8 cm, chest fusion, two hearts with a fetal heartbeat, abdominal fusion, two bladders, two spines with abnormal physiological curvature, four upper limbs, and four lower limbs (Fig. 1A). The couple had no family history of congenital anomalies.

The staffs of multidisciplinary team extensively counseled the couple regarding the treatment and prognosis of the conjoined twins. The parents chose selective termination of the conjoined twins. Thus, intrathoracic injection of potassium chloride (KCl) to the conjoined twins was performed under an ultrasound-guided procedure at 16 weeks of gestation. Images of the live fetus and conjoined twins after selective termination are shown in Fig. 1B. The couple refused chromosome examination in conjoined twins, and amniocentesis was performed on the other fetus. The result of chromosome microarray analysis in the other fetus was normal.

The woman was followed up closely. Cesarean section was performed due to central placenta previa at 37 weeks. The healthy female baby weighed 3270 g with Apgar scores of 9 and 10 at the first and fifth minute, respectively, whereas the papyraceous conjoined fetuses weighed 51 g (Fig. 1C). The baby is now 1 year and 5 months old, and she is in good health.

### Case 2

**Fig. 2:**
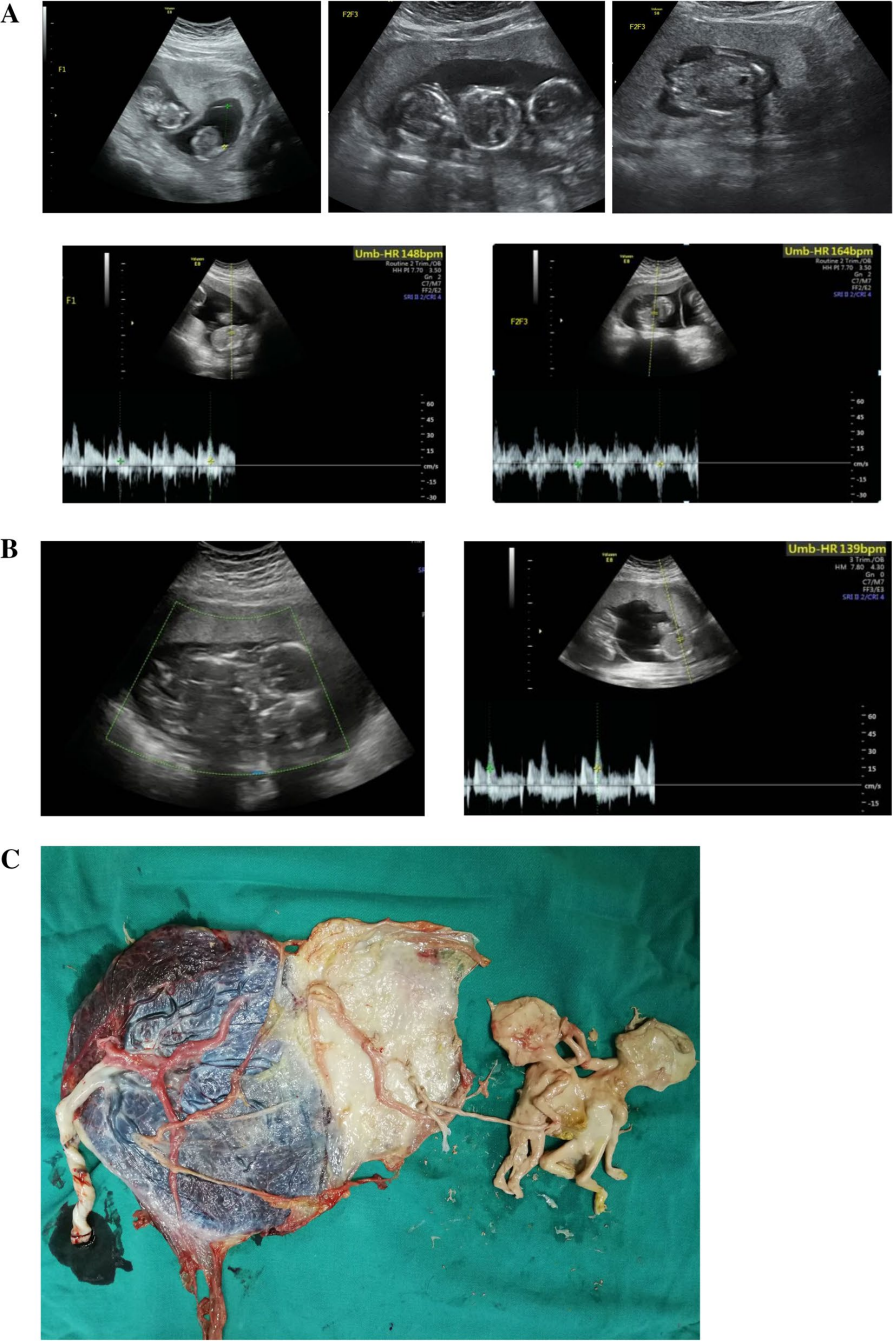
**A.** Images of conjoined twins (F2 and F3) of case 2. **B.** Images of live fetus (F1) and conjoined twins after selective termination of case 2. **C.** Images of the dichorionic-diamniotic placenta and the papyraceous conjoined twins of case 2

A 22-year-old woman, gravida 1, para 0, underwent in vitro fertilization and embryo transfer. At 13 weeks and 5 days of gestation, ultrasound examination showed a DCDA triplet pregnancy with thoraco-omphalopagus conjoined twins. The chest and abdominal wall of the conjoined twins were connected, and only one heart echo was found. The livers of the two fetuses were connected, and the limbs were independent. Independent gastric vesicles and the spinal echoes of each fetus could be found (Fig. 2A). The couple had no family history of congenital anomalies.

After extensive counsel by the multidisciplinary team, selective termination of the conjoined twins was chosen by the couple. Thus, ultrasound-guided intrathoracic injection of KCl at 17 weeks of gestation was performed on the conjoined twins. Images of the live fetus and conjoined twins after selective termination are shown in Fig. 2B. The couple was only willing to perform amniocentesis in the other fetus, not the conjoined twins. The result of chromosome microarray analysis in the other fetus was normal.

At 37 weeks and 4 days, a healthy female baby weighing 2760 g was delivered with Apgar scores of 10 and 10 at 1 and 5 min, respectively, whereas the papyraceous conjoined fetuses weighed 79 g (Fig. 2C). The baby is now 1 year and 4 months old, and she is in good health.

### Case 3

**Fig. 3:**
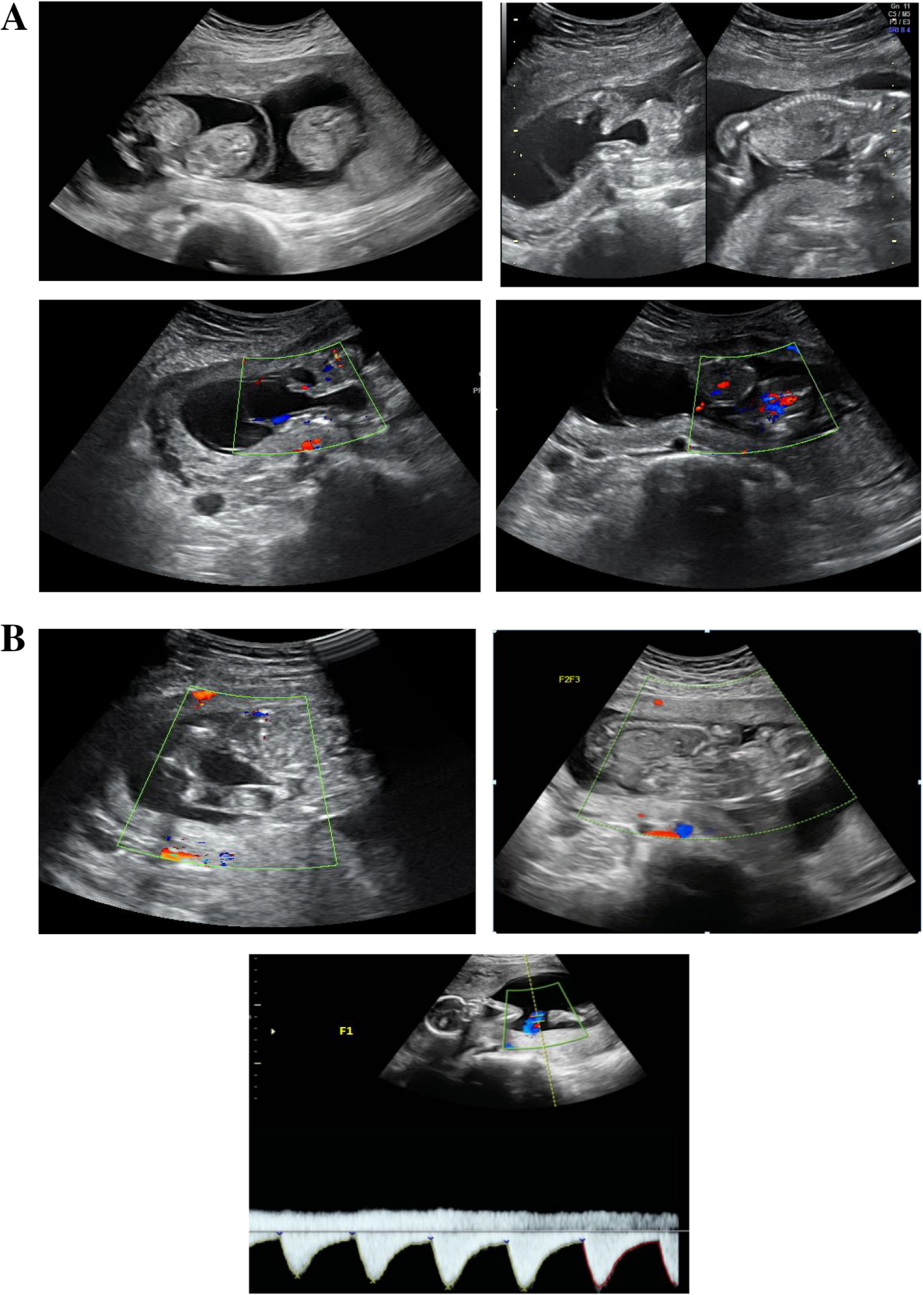
**A.** Images of conjoined twins (F2 and F3) of case 3. **B.** Images of live fetus (F1) and conjoined twins after selective termination of case 3

A 29-year-old woman, gravida 3, para 1, conceived spontaneously. Due to the suspicion of omphalopagus conjoined twins in a triplet pregnancy, she was transferred to our department at 14 weeks, and ultrasound examination in our hospital showed omphalopagus conjoined twins in a DCDA triplet pregnancy. In the conjoined twins, there was a 4.7 × 3.5 × 4.5 cm cystic space in the amniotic cavity of the conjoined twins, which was connected to the two bladders of conjoined twins. Only one allantoic artery could be seen on the surface of both fetal bladders, and a urachal cyst was suspected to be present (Fig. 3A). The couple had no family history of congenital anomalies.

The staffs of multidisciplinary team extensively counseled the couple. Based on the couple’s choice, selective termination of the conjoined twins was performed by ultrasound-guided intrathoracic injection of KCl at 15 weeks and 2 days of gestation, and amniocentesis was done in the other fetus, not in the conjoined twins. Images of the live fetus and conjoined twins after selective termination are shown in Fig. 3B. The result of chromosome microarray analysis in the living fetus was normal.

At 33 weeks and 6 days, a healthy female baby weighing 2450 g was delivered due to suspected fetal distress, with Apgar scores of 8 and 10 at 1 and 5 min, respectively, whereas the papyraceous conjoined fetuses weighed 84 g. The baby is now one month old, and she is in good health.

## Discussion and conclusions

Conjoined twins are a rare and serious complication of monochorionic twins. The total incidence is 1.5 per 100,000 births, and about 50% are liveborn [1]. Conjoined twins are more common in females, and the male-to-female ratio is 1:3 [4]. The pathogenesis of conjoined twins is unclear. Fission theory [5] and fusion theory [6] are widely accepted. The fission theory suggests that the embryo undergoes incomplete division 13–15 days after fertilization, resulting in conjoined twins. The fusion theory suggests that two separate embryos undergo a second fusion 13 days after fertilization.

Conjoined twins can be classified according to their most prominent conjoined parts. There are many classifications of conjoined twins. Broadly speaking, it can be divided into dorsally conjoined twins and nondorsally conjoined twins [6]. According to Spencer’s classification [7], there are eight types of conjoined twins as follows: (1) cephalogapus, (2) thoracopagus, (3) omphalopagus, (4) ischiopagus, (5) parapagus, (6) craniopagus, (7) pygopagus, and (8) rachipagus. However, many conjunction types show overlapping conjunction patterns, leading to the various phenotypes of conjoined twins. Thoracoomphalopagus is one of the most common types of conjoined twins with a rate of 75% [3].

Prenatal screening and diagnosis of conjoined twins is usually performed by ultrasonography [4]. Characteristics of prenatal ultrasound diagnosis of conjoined twins include [4, 8]: (1) a single placenta without amniotic septum, (2) fetuses lying in the same constant position with heads and body parts at the same level persistently, (3) inseparable body and skin contours, (4) fetuses facing each other with hyperflexion of cervical spines, sharing of organs, and a single umbilical cord with more than three vessels, (5) fewer limbs of some conjoined twins than that of normal twins, and (6) abnormal flexion of the spine. MRI can be used to assist in the diagnosis if necessary [9].

Conjoined twins have a low survival rate, and the prognosis is generally poor. Twenty-five percent of live births live to the age of surgery [5]. Only 60% of surgical separation cases survive [10]; therefore, early antenatal diagnosis is important. The prognosis is mainly affected by specific fusion parts and related malformations. Due to the high incidence of complex heart defects, the survival rate of thoracopagus is the lowest [11]. Elective separation is feasible in omphalopagus, pygopagus, and some craniopagus and thoracopagus twins. Separation is not possible in cephalopagus, parapagus, and rachipagus twins [1]. The difficulty and prognosis of surgical treatment are related to the location and degree of conjoined parts. The separation of conjoined twins is complex and expensive, involving multidisciplinary teams. When separation involves the unequal sharing of limbs and organs, or when separation leads to the death of one of the conjoined twins, complex ethical issues will also arise [1].

In clinical management, excluding the severity of the case, parents’ social situation, religious, and psychological beliefs should be considered.

In triplet pregnancies, one should be aware about the possibility of conjoined twins. Triplet pregnancies contribute significantly to maternal complications, including spontaneous abortion, preterm birth, gestational diabetes, hypertensive disorders of pregnancy, antepartum bleeding, anemia, hyperemesis gravidarum, cesarean section, and postpartum hemorrhage. In addition, women delivering triplets have a significantly higher risk of cardiac disease and acute or chronic lung disease compared to women who give birth to twins [12, 13]. Compared with singletons and twins, triplets have a higher risk of adverse perinatal outcomes due to higher rates of preterm birth, low birth weight, and congenital anomalies [14, 15]. Therefore, neonatal morbidity and mortality rates may also increase. The risk of spontaneous loss of the pregnancy prior to 24 weeks is 15–18% for triplets and 8% for twins [16, 17] and preterm delivery prior to 34 weeks is 50%, while approximately 8–17% of triplets are delivered between 24 and 28 weeks [18, 19]. In triplets, the rate of cerebral palsy is 28 per 1000 live births, compared to 1.6 per 1000 live births in singletons and 7 per 1000 live births in twin pregnancies. The infant mortality rate in triplets is 52.5 per 1000 live births, compared to 5.4 per 1000 live births in singletons and 23.6 per 1000 live births in twins [20].

Conjoined twins in triplet pregnancy are considered a unique phenomenon that is accompanied by a wide variety of congenital abnormalities and has hazardous consequences for both fetuses and parents, which also occurs in monochorionic diamniotic (MCDA) triplets and DCDA triplets. Due to its rarity, triplet pregnancies with increased maternal complications, perinatal morbidity and mortality, provide a great challenge for staff to undertake the complete workup, determine shared anatomy, evaluate maternal-fetal prognosis, and decide management (to continue as a triplet gestation, termination of pregnancy, or selective termination of conjoined twins) once a diagnosis is reached. Up to now, no consensus has been achieved.

We used a list of keywords including “conjoined twins,” “triplet pregnancy,” “dichorionic diamniotic,” “monochorionic”, and “multiple pregnancy” to perform an extensive Medline and CNKI search of the literature in English and Chinese about the perinatal management and outcomes of conjoined twins in triplet pregnancies. To the best of our knowledge, the number of published papers related to conjoined twins in triplet pregnancies was less than 30. There were four reported cases of unclear chorionic triplet pregnancies [21–24]. Conjoined twins all had a poor prognosis. Detailed information is shown in Table 1. We found 14 cases of monochorionic triplet pregnancies with conjoined twins [25–38]. Detailed information is shown in Table 2. Due to placental vascular anastomoses between the conjoined twins and the other fetus, the prognosis of the normal triplet was poor after selective fetal reduction. We also found 10 cases of conjoined twins in dichorionic diamniotic triplet pregnancies [3, 31, 39–46]. Detailed information is shown in Table 3. Compared with conjoined twins in monochorionic triplet pregnancies, the prognosis in dichorionic diamniotic triplet pregnancies is generally better. Except for two cases, all cases achieved good pregnancy outcomes after selective reduction.

**Table 1:** Reported cases of conjoined twins with unclear chorionic triplet pregnancy

Study ID	Maternal age (years)	Mode of conception	Triplet type	Diagnosis age (wks)	Type of conjoining	Prenatal intervention of conjoined twins	Intervention weeks	Delivery age (wks)	Delivery method	Outcome of the fetuses	
										Conjoined twins	Single fetus
Hartung RW 1984 [21]	21	NS	NS	NS	Cranio-thoraco-omphalopagus	No	/	NS	CS	Live birth weighing 1140 g and died 1 h later	Live birth weighing 950 g
Koontz WL 1985 [22]	32	NS	NS	29	Cranio-thoraco-omphalopagus	No	/	31	CS	Live birth weighing 1580 g and died shortly after birth	Live birth weighing 1390 g
Apuzzio JJ 1988 [23]	30	Spontaneous	NS	15	Thoraco-omphalopagus	No	/	NS	TOP	/	/
Kaveh M 2013 [24]	27	Spontaneous	NS	15	Thoraco-omphalopagus	No	/	36	CS	Live birth weighing 3080 g and died 5 days later	Live birth no weight data

*NS* not specified, *wks* weeks, *CS* cesarean section, *TOP* termination of pregnancy

**Table 2:** Reported cases of monochorionic triplet pregnancy with conjoined twins

Study ID	Maternal age (years)	Mode of conception	Triplet type	Diagnosis age(wks)	Type of conjoining	Prenatal intervention of conjoined twins	Intervention weeks	Delivery age(wks)	Delivery method	Outcome of the fetuses	
										Conjoined twins	Single fetus
Tan KL 1971 [25]	22	Spontaneous	MCDA	39	Thoraco-omphalopagus	No	/	39	CS	Live birth weighing 4765 g and died a few minutes later	Live birth weighing 2870 g
Lipitz S 1995 [26]	25	Spontaneous	MCDA	16	Thoracopagus	Selective fetal reduction by intracardiac injection of potassium chloride	16+	16+	Induced labor due to intrauterine demise of normal single triplet	/	IUD a few hours later after prenatal intervention
Chang DY 1996 [27]	26	Spontaneous	MCMA	24+	omphalopagus	No	/	25	Induced labor due to intrauterine demise of the fetuses	IUD weighing 1240 g	Acardiac fetus weighing 80 g
Gardeil F 1998 [28]	34	Spontaneous	MCDA	13	Thoraco-omphalo-ischiopagus	No	/	36	CS	Live birth weighing 2900 g and died 6 months later	Live birth weighing 2041 g
Wax JR 1999 [29]	18	Spontaneous	MCMA	16 + 5	Thoracopagus	No	/	/	TOP	/	/
Zeng SM 2002 [30]	22	Spontaneous	MCDA	22	Thoracopagus	No	/	32	CS	Live birth, no weight data, And died 35 days later due to cardiopulmonary collapse	Live birth,no weight data
Sepulveda W 2003 [31]	41	Spontaneous	MCDA	13	Cephalopagus	Selective fetal reduction by endoscopic laser occlusion of the single umbilical cord	16	28	CS due to intrauterine demise of normal single triplet at 28 weeks	/	Dead fetus weighing 1010 g
Suzumori N 2006 [32]	33	Spontaneous	MCDA	13	Cephalopagus	No	/	/	TOP	/	/
Sellami A 2013 [33]	20	Spontaneous	MCDA	21	Xipho-omphalopagus	No	/	/	TOP	/	/
Talebian M 2015 [34]	38	IVF-ET	MCDA	12 + 2	Thoraco-omphalopagus	Selective fetal reduction by radiofrequency ablation	16	17	Spontaneous abortion due to PROM	Death no weight data,	Death no weight data,
Yuan HX 2017 [35]	39	IVF-ET	MCDA	10	Thoraco-omphalopagus	No	/	/	TOP	/	/
Mariona F 2017 [36]	26	Spontaneous	MCDA	9 + 2	Parapagus	No	/	34	CS	Live birth weighing 2485 g and died 32 min later	Live birth weighing 3175 g
Meng XL 2018 [37]	36	Spontaneous	MCDA	13 + 5	Thoraco-omphalopagus	Selective fetal reduction by microwave ablation	16	17	Induced labor due to intrauterine demise of normal single triplet at 17 weeks	/	IUD at 17 week
Gao QQ 2021 [38]	26	Spontaneous	MCDA	12	Omphalopagus	No	/	15	Induced labor due to intrauterine demise of three fetuses at 15 weeks	IUD	IUD

*wks* weeks, *NS* not specified, *MCDA* monochorionic diamniotic, *CS* cesarean section, *IVF-ET* in vitro fertilization and embryo transfer, *MCMA* monochorionic monoamniotic, *TOP* termination of pregnancy, *PROM* premature rupture of membrane

**Table 3:** Reported cases of dichorionic diamniotic triplet pregnancy with conjoined twins

Study ID	Maternal age (years)	Mode of conception	Triplet type	Diagnosis age(wks)	Type of conjoining	Prenatal intervention of conjoined twins	Intervention weeks	Delivery age(wks)	Delivery method	Outcome of the fetuses	
										Conjoined twins	Single fetus
Skupski DW 1995 [39]	35	IVF-ET	DCDA	12	Thoraco-omphalopagus	Selective fetal reduction by intracardiac injection of KCl	12+	NS	NS	/	Pregnancy to the third trimester
Goldberg Y 2000 [40]	28	IVF-ET	DCDA	8 + 4	Thoraco-omphalopagus	Selective fetal reduction by intracardiac injection of KCl	12+	NS	NS	/	Pregnancy was ongoing
Timor-Tritsch IE 2000 [41]	32	IVF-ET	DCDA	10	Omphalopagus	Selective fetal reduction by intracardiac injection of KCl	10 + 3	NS	NS	/	Live birth no weight data
Sepulveda W 2003 [31]	29	Spontaneous	DCDA	10 + 3	Thoracopagus	No	/	38	CS	IUD at 12 wks	Live birth weighing 2740 g
Charles A 2005 [42]	NS	IVF-ET	DCDA	10	Omphalopagus	Selective fetal reduction	15	21	NS	/	no weight data Death due to premature delivery
Hirata T 2009 [43]	34	IVF-ET	DCDA	8	Thoracopagus	No	/	39	VD	IUD at 10 + 3 wks	Live birth weighing 2792 g
Shepherd LJ 2011 [44]	32	ovulation induction	DCDA	13 + 1	Thoraco-omphalopagus	Selective fetal reduction by intracardiac injection of KCl	13 + 5	40	VD	/	Live birth weighing 3590 g
Takae S 2011 [45]	28	NS	DCDA	19	Thoracopagus	No	/	33	CS	Live birth weighing 2767 g and died 23 days later	Live birth weighing 1754 g
Ozcan HC 2017 [3]	28	Spontaneous	DCDA	17	Thoraco-omphalopagus	Selective fetal reduction by intracardiac injection of KCl	21	21 + 1	CS	/	IUD at 21 + 1 wks
Castro PT 2017 [46]	32	IVF-ET	DCDA	9	Thoraco-omphalopagus	No	/	38	VD	IUD at 13 wks	Live birth weighing 2750 g
Our cases	44	Spontaneous	DCDA	12	Cranio-thoraco-omphalopagus	Selective fetal reduction by intracardiac injection of KCl	16	37	CS	weighing 51 g	Live birth weighing 3270 g
	22	IVF-ET	DCDA	13 + 5	Thoraco-omphalopagus	Selective fetal reduction by intracardiac injection of KCl	17	37 + 4	CS	weighing 79 g	Live birth weighing 2760 g
	29	Spontaneous	DCDA	14	Omphalopagus	Selective fetal reduction by intracardiac injection of KCl	15 + 2	33 + 6	CS	weighing 84 g	Live birth weighing 2450 g

*wks* weeks, *NS* not specified, *CS* cesarean section, *VD* vaginal delivery, *IVF-ET* In vitro fertilization and embryo transfer, *KCl* potassium chloride, *TOP* termination of pregnancy, *PROM* Premature rupture of membrane

Over other studies, the advantage of our study is that we reported three cases of conjoined twins in DCDA triplet pregnancy, and detailed information about maternal, fetal, and neonatal status is provided in our study. Due to the poor prognosis of conjoined twins evaluated by multidisciplinary teams, conjoined twins were selectively terminated by transabdominal intracardiac potassium chloride injection in the second trimester. Among our three cases, the pregnant women were stable throughout the pregnancy, two cases with term delivery (case 1 and case 2), one case with preterm delivery at 33 weeks and 6 days of gestation due to suspected fetal distress. All the cases have a good prognosis, and the three babies were followed up and are currently in good health.

This study reviewed the published papers about perinatal outcomes on conjoined twins in DC and MC triplet pregnancies and gives three new cases in the selective termination of the conjoined twins and the outcome of normal triplets in DCDA triplets, which may be useful for making clinical decisions in triplet pregnancies with conjoined twins. The limitation of this study is the loss of some data in the literature review due to the lack of information in the published papers.

In conclusion, due to the rarity of triplet pregnancies with conjoined twins, experience with the treatment is limited. Obstetricians and ultrasound specialists must be aware of the rare complications and focus on early ultrasound diagnosis. Early antenatal diagnosis of conjoined twins and determination of chorionicity of triplet gestations are critical for individualized management options and the prognosis of the normal fetus. Expecting parents should be extensively counseled by a multidisciplinary team. Early selective termination of the conjoined twins by intrathoracic injection of potassium chloride may be a procedure in dichorionic diamniotic triplet pregnancies to improve the perinatal outcomes of the normal fetus in triplets.

## Data Availability

The datasets used and/or analyzed during the current study are available from the corresponding author upon reasonable request.

## Abbreviations

DCDA: Dichorionic diamniotic
MCDA: Monochorionic diamniotic
KCl: Potassium chloride
